# MCP‐1 expression in breast cancer and its association with distant relapse

**DOI:** 10.1002/cam4.6284

**Published:** 2023-06-21

**Authors:** Bridie S. Mulholland, Pierre Hofstee, Ewan K. A. Millar, Dana Bliuc, Sandra O'Toole, Mark R. Forwood, Michelle M. McDonald

**Affiliations:** ^1^ Graduate School of Medicine, Faculty of Science, Medicine and Health University of Wollongong Wollongong New South Wales Australia; ^2^ Susan Wakil School of Nursing and Midwifery, Faculty of Medicine and Health University of Sydney Camperdown New South Wales Australia; ^3^ The Tweed Hospital Northern New South Wales Local Health District Tweed Heads New South Wales Australia; ^4^ St George and Sutherland Clinical Campuses, School of Clinical Medicine UNSW Medicine and Health, University of New South Wales Sydney New South Wales Australia; ^5^ Department of Anatomical Pathology, NSW Health Pathology St George Hospital Kogarah Australia; ^6^ Translational Breast Cancer Research Group, Cancer Ecosystems Program Garvan Institute of Medical Research Sydney New South Wales Australia; ^7^ Bone Microenvironment Group, Skeletal Diseases Program Garvan Institute of Medical Research Sydney New South Wales Australia; ^8^ Department of Tissue Pathology and Diagnostic Pathology Royal Prince Alfred Hospital Camperdown New South Wales Australia; ^9^ Sydney Medical School, Faculty of Medicine and Health University of Sydney Camperdown New South Wales Australia; ^10^ School of Pharmacy and Medical Sciences Menzies Health Institute Queensland, Griffith University Gold Coast Queensland Australia; ^11^ School of Medical Sciences, Faculty of Medicine and Health University of Sydney Camperdown New South Wales Australia

**Keywords:** CCL2, metastasis, neoplasm, protein, tumour

## Abstract

**Background:**

Distant relapse of breast cancer complicates management of the disease and accounts for 90% of breast cancer‐related deaths. Monocyte chemoattractant protein‐1 (MCP‐1) has critical roles in breast cancer progression and is widely accepted as a pro‐metastatic chemokine.

**Methods:**

This study explored MCP‐1 expression in the primary tumour of 251 breast cancer patients. A simplified ‘histoscore’ was used to determine if each tumour had high or low expression of MCP‐1. Patient breast cancers were retrospectively staged based on available patient data. *p* < 0.05 was used to determine significance and changes in hazard ratios between models were considered.

**Results:**

Low MCP‐1 expression in the primary tumour was associated with breast cancer‐related death with distant relapse in ER− breast cancers (*p* < 0.01); however, this was likely a result of most low MCP‐1‐expressing ER− breast cancers being Stage III or Stage IV, with high MCP‐1 expression in the primary tumour significantly correlated with Stage I breast cancers (*p* < 0.05). Expression of MCP‐1 in the primary ER− tumours varied across Stage I, II, III and IV and we highlighted a switch in MCP‐1 expression from high in Stage I ER− cancers to low in Stage IV ER− cancers.

**Conclusion:**

This study has emphasised a critical need for further investigation into MCP‐1's role in breast cancer progression and improved characterisation of MCP‐1 in breast cancers, particularly in light of the development of anti‐MCP‐1, anti‐metastatic therapies.

## Graphical abstract

The role of MCP‐1 in breast cancer and its use as a predictive tool remains conflicting. This study shows that MCP‐1 expression in primary ER− breast tumours is varied across the four breast cancer stages in an Australian cohort of women.

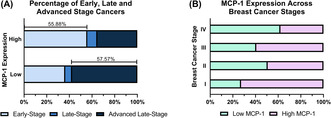

## 1 INTRODUCTION

The formation of metastases is a considerable clinical obstacle that prevents the successful management of cancer. Breast cancer is the most common cancer experienced by women. Approximately 30% of women diagnosed with early‐stage breast cancer will progress to advanced disease within 10 years post‐diagnosis, with 10%–15% of patients developing distant metastases within 3 years.^1^ Approximately 90% of deaths from breast cancer are related to metastatic disease. The bone, liver, lungs and brain are the most frequent sites of breast cancer metastasis; bone being the most frequent site, occurring in 70% of all metastatic cases.^2^, ^3^

Heterogeneity of breast cancers severely complicates treatment of the disease and is responsible for variability in clinical behaviour and response to treatment. Breast cancer morphology and biology is varied and, thus, clinical behaviour and treatment‐response also varies.^4^ Typing, grading and staging of the primary tumour comprise the classical assessment of breast cancer. Typing refers to the histological classification, grading refers to the microscopic assessment of histological differentiation and staging refers to the classification of the cancer into one of five stages–0, I, II, III and IV–based on tumour size, nodal status and the presence of distant metastases.^4^ Furthermore, breast cancers can be classified using surrogate immunohistochemistry markers, namely oestrogen receptor (ER), progesterone receptor (PR), human epidermal growth factor receptor 2 (HER2) and antigen KI‐67 (Ki‐67).^5^

Monocyte chemoattractant protein‐1 (MCP‐1) is a chemotactic protein that has been widely implicated in breast cancer progression and metastasis as a pro‐progression and pro‐metastatic chemokine, and is a promising target for anti‐metastatic therapies.^6^ In terms of breast cancer progression, as a chemotactic protein, MCP‐1 most notably associates with infiltration and recruitment of inflammatory cells, particularly macrophages,^7^, ^8^, ^9^, ^10^ tumour‐associated macrophages,^11^ monocytes^12^ and mesenchymal stem cells.^13^ Targeted gene silencing of MCP‐1 effectively inhibits triple negative breast cancer progression by blocking the recruitment of M2 macrophages and cancer stem cell renewal in mice.^8^

Subsequent to its involvement in inflammatory cell infiltration and recruitment, high levels of MCP‐1 have been associated with stroma development,^11^, ^14^ angiogenesis^15^, ^16^ and metastasis.^17^, ^18^, ^19^ Dutta et al.^19^ showed that MCP‐1 is overexpressed in basal‐like cell lines and that its overexpression drives invasiveness and metastasis. Human studies into MCP‐1's role in breast cancer progression are more conflicted. Lebrecht et al.^20^ concluded that in patients with breast cancer, elevated MCP‐1 was associated with advanced disease; yet, Dehqanzada et al.^21^ showed that high MCP‐1 levels were associated with favourable prognostic variables. A meta‐analysis conducted in 2014 concluded that MCP‐1 is a poor diagnostic and prognostic marker for solid tumours.^22^ However, MCP‐1's specific utility as a marker of metastatic potential for breast cancer has not been readily explored.

This study explored the expression of MCP‐1 in breast tumour samples from a cohort of 251 Australian women with breast cancer and examined whether MCP‐1 expression is correlated with incidence of metastasis–termed distant relapse in this study–and survival. Given the overexpression of MCP‐1 in basal‐like breast cancers that are ER−, we hypothesised that high MCP‐1 expression in the primary tumour of ER− breast cancers would be correlated with a greater incidence of distant relapse and decreased survival.

## 2 METHODS

MCP‐1 expression was assessed by immunohistochemical (IHC) staining of tissue microarrays (TMAs) of formalin‐fixed, paraffin‐embedded blocks containing samples of breast tumour tissue from a cohort of 251 patients diagnosed with invasive ductal breast carcinoma between February 1992 and August 2002 at St Vincent's Public and Private hospitals in Sydney, Australia.^23^ Prior approval for this study was obtained from the St Vincent's Hospital Human Research Ethics Committee (HREC SVH H94/080; HREC 06336 SVH H00 036) and all study participants gave informed consent for their inclusion in the study.

### 2.1 Clinical characteristics of cohort

Patient characteristics are described in detail by Millar et al.^23^ Follow‐up ranged between 0 and 152 months with a median follow‐up of 64 months. Distant relapses and metastases were defined as disease in the lungs, liver, brain or distant lymph nodes.^23^ Two patients were unable to be analysed due to missing tumour cores, bringing the total sample size to 249. Patient age ranged from 32 to 79 with a median age of 55.

### 2.2 TMA preparation and scoring

Antigen retrieval and staining was performed using the Leica Biosystems BOND RX Research Stainer (Leica Microsystems). Antigen retrieval was performed using the BOND Epitope Retrieval Solution 2 (pH 9.0; Leica Biosystems, catalogue no. AR9640) and staining for MCP‐1 was performed using the Anti‐MCP1 Antibody (Abcam, catalogue no. ab73680) at a dilution of 1 in 500. Slides were counterstained with haematoxylin. Adrenal tissue was used as a positive control for staining optimisation in accordance with the manufacturer's recommendation. All IHC staining was performed by the Garvan Histopathology Facility.

MCP‐1 staining was scored using QuPath image analysis software,^24^ supervised by a pathologist blinded to group affiliation. Briefly following de‐arraying of the TMA cores and automated estimation of stain vectors for the Haematoxylin DAB stained IHC slides, intensity parameters were set to differentiate positive cytoplasmic staining as weak (1+), moderate (2+) or strong (3+). A machine learning tissue classifier (random trees) was then trained using selected annotations to classify cells into cancer epithelium or stromal classes. Once satisfactory performance was achieved the algorithm was run over all slides in one batch and results obtained. The QuPath values for percentage of MCP‐1‐positive carcinoma cells and intensity of cytoplasmic staining were multiplied to derive a simplified ‘histoscore’. The mean histoscore for each patient was used for analysis. Low MCP‐1 expression included patients with histoscores less than the median histoscore; high MCP‐1 expression included patients with histoscores higher than the median histoscore. The median histoscore was 97.3 (range = 15.3–286; interquartile range [IQR] = 37.0).

### 2.3 Retrospective breast cancer scoring

ER− breast cancers were retrospectively staged based on available patient data. Patients were identified as having Stage I, II, III or IV ER− breast cancer in accordance with the 8th edition of the American Joint Committee on Cancer's TNM cancer staging system.^25^

### 2.4 Statistical analyses

The Kaplan–Meier method was employed to estimate the likelihood of distant relapse and breast‐cancer related death with distant relapse in patients with primary tumours of low MCP‐1 expression or high MCP‐1 expression. To compare survival between the two expression groups, the log‐rank test was used. Hazard ratios (HRs) and associated 95% confidence intervals were examined using the Cox proportional hazard regression model to adjust for potential confounding variables. Potentially confounding variables from the available patient date were identified as: age, use of chemotherapy, grade of primary tumour and lymph node involvement. For this study, age was kept as a continuous variable. Unit comparisons for each variable were as follows: age–per 1 year; MCP‐1—low expression versus high expression; grade—primary tumour grade of two versus primary tumour grade of 3; chemotherapy—yes patient received chemotherapy versus no patient did not receive chemotherapy; lymph node involvement–no patient did not have lymph node involvement versus yes patient had lymph node involvement. Univariate, age‐adjusted and multivariable‐adjusted models were generated. *p* < 0.05 was used to determine significance and changes in HRs between models were considered. Chi‐squared analysis was used to determine correlations between MCP‐1 expression and clinical characteristics, and MCP‐1 expression and breast cancer stages. Kaplan–Meier and chi‐squared analysis were conducted in GraphPad Prism 8 (version 8.4.3; GraphPad Software). Cox proportional hazards models were generated using SPSS (version 1.0.0.1447; International Business Machines Corporation).

## 3 RESULTS

A total of 180 patients were identified as having ER+ breast cancer, with the remaining 69 patients identified as having ER− breast cancer. The ER+ breast cancer group included patients with luminal A and luminal B cancers, the ER− breast cancer group included patients with basal‐like, HER2+ In total, 66 patients experienced distant relapse and 49 of those patients with distant relapse died from breast cancer‐related causes within a median follow‐up period of 64 months.

### 3.1 Clinical characteristics of cohort and association with MCP‐1 expression

The relationship between MCP‐1 expression and clinical characteristics is described in Table 1. Age, oestrogen receptor status, grade, lymph node involvement, the use of endocrine therapy and the use of chemotherapy were similar between groups.

**TABLE 1:** Clinical characteristics of the study cohort.

Variable	Low MCP‐1 (*n* = 123)	High MCP‐1 (*n* = 124)	*p*‐value
Age (years), mean (range)	54.90 (59.7)	55.4 (56.5)	NS
Oestrogen receptor status, *n* (%)			NS
Positive	90 (71.79)	90 (71.90)	
Negative	33 (28.21)	34 (28.10)	
Tumour size (mm), median (IQR)	20.0 (13.5)	17.5 (12.0)	NS
Grade (primary tumour), *n* (%)			NS
1	22 (17.89)	15 (12.10)	
2	43 (34.96)	50 (40.32)	
3	58 (47.15)	59 (50.43)	
Lymph node involvement, *n* (%)	59 (48.36)	54 (43.90)	NS
Treatment, *n* (%)			
Endocrine therapy	59 (47.20)	66 (53.23)	NS
Chemotherapy	48 (39.02)	52 (41.94)	NS
Distant relapse, *n* (%)	86 (52.5)	95 (47.5)	NS

*Note*: Age and tumour size were analysed using a linear regression. Grade, lymph node involvement, treatment and distant relapse were analysed by Chi‐squared testing.

Abbreviations: IQR, interquartile range; MCP‐1, monocyte chemoattractant protein‐1; NS, not significant.

### 3.2 Immunohistochemical analysis of MCP‐1 expression in invasive ductal carcinoma

**FIGURE 1:**
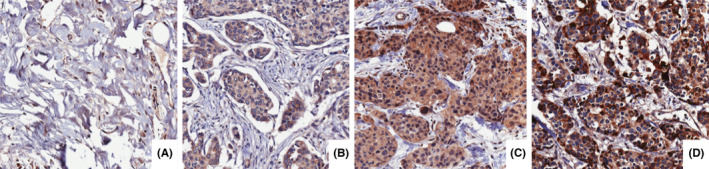
Representative immunohistochemical staining intensities at 20× magnification. (A) represents normal breast tissue; (B) represents 1+ cytoplasmic staining intensity in invasive breast carcinoma epithelium; (C) represents 2+ cytoplasmic staining intensity in invasive breast carcinoma epithelium; (D) represents 3+ cytoplasmic staining intensity in invasive breast carcinoma epithelium.

Representative IHC staining intensities of MCP‐1 are illustrated in Figure 1. The staining pattern for MCP‐1 was cytoplasmic with 100% of tumour samples showing evidence of MCP‐1 staining. Staining intensity ranged from 1+ to 3+, with the percentage‐of‐positive‐tumour cells ranging from 30% to 100%.

### 3.3 Low MCP‐1 expression in primary tumour associated with increased risk of breast cancer‐related death with distant relapse in ER− breast cancers but not ER+ breast cancers

**FIGURE 2:**
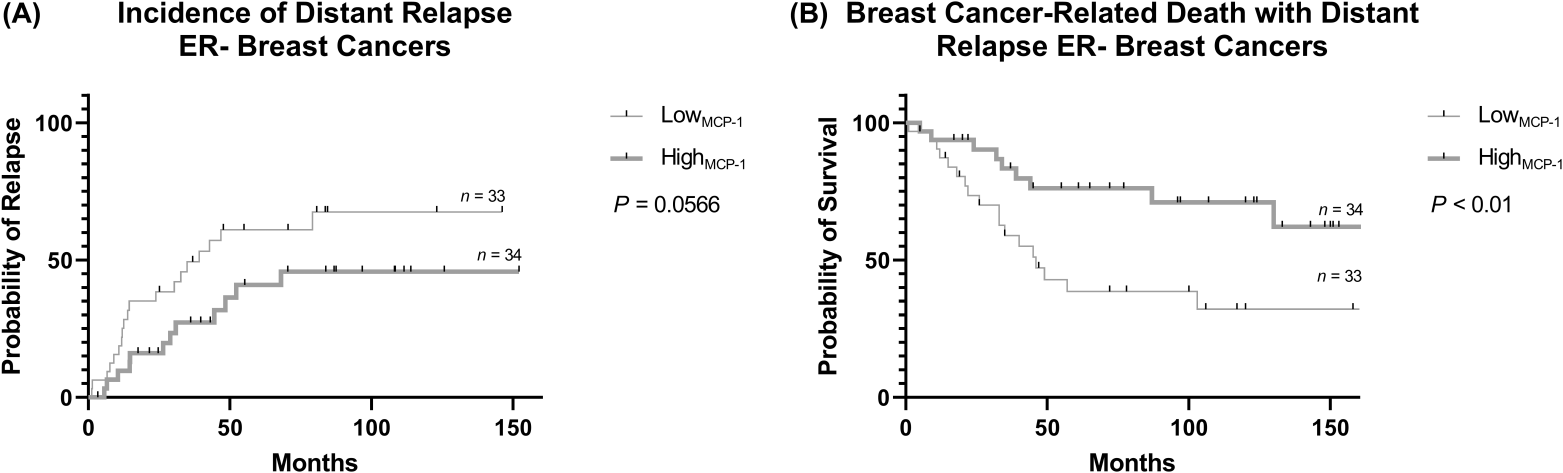
Kaplan–Meier curve describing probability of distant relapse and breast cancer‐related death with distant relapse in patients with high and low expression of MCP‐1 in primary ER‐ breast tumours. The log‐rank test was used to compare probability between groups. Censored patients are represented as solid black lines. ER, oestrogen receptor; MCP‐1, monocyte chemoattractant protein‐1; (−), negative.

**FIGURE 3:**
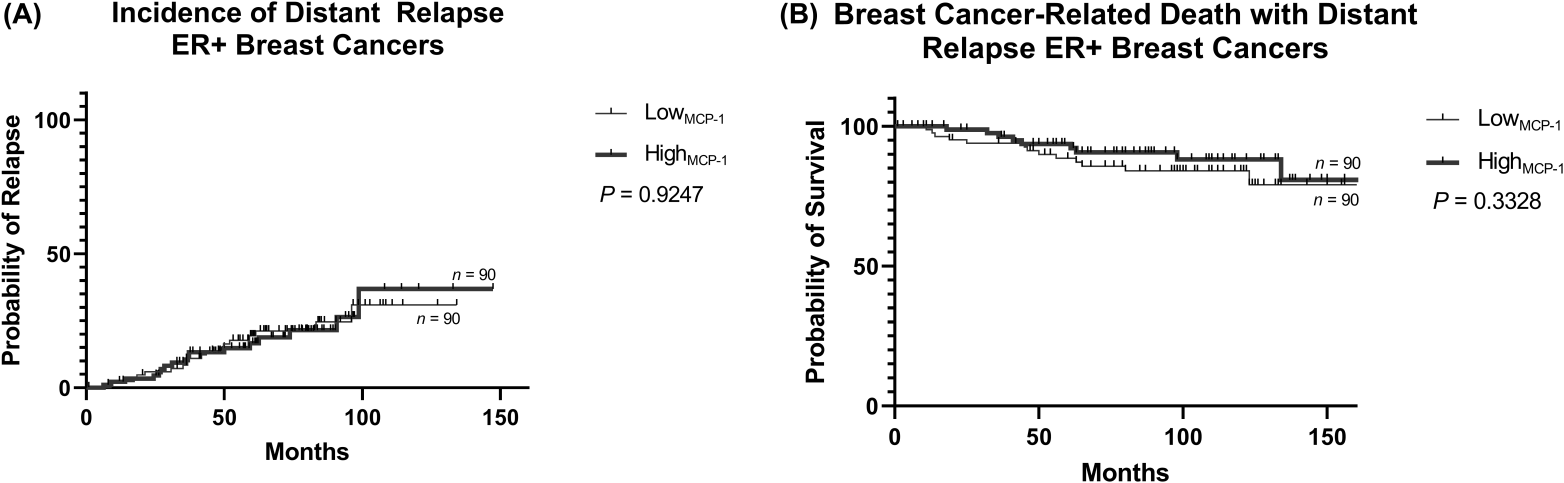
Kaplan–Meier curve describing probability of distant relapse and breast cancer‐related death with distant relapse in patients with high and low expression of MCP‐1 in primary ER+ breast tumours. The log‐rank test was used to compare probability between groups. Censored patients are represented as solid black lines. ER, oestrogen receptor; MCP‐1, monocyte chemoattractant protein‐1; (+), positive.

The Kaplan–Meier method was employed to estimate the likelihood of distant relapse in low MCP‐1 and high MCP‐1 groups. Comparison of curves for low MCP‐1 and high MCP‐1 groups showed that patients with ER− breast cancers with low MCP‐1 expression were at increased risk of breast cancer‐related death with distant relapse (*p* < 0.01; Figure 2B). MCP‐1 expression was not associated with risk of distant relapse and breast cancer‐related death with distant relapse in ER+ breast cancers (Figure 3).

### 3.4 Cox proportional hazards models for determination of confounding variables

Cox proportional hazards models were generated to assess if MCP‐1 acted as an independent prognostic variable or if it was confounded by other variables. MCP‐1 and lymph node involvement maintain the highest HRs across univariate, age‐adjusted and multivariable‐adjusted models for distant relapse (MCP‐1: adjusted HR = 1.66; 95% CI = 0.77–3.61, lymph node involvement: adjusted HR = 4.91; 95% CI = 2.09–11.56) (Table 2) and breast cancer‐related death with distant relapse (MCP‐1: adjusted HR = 2.21; 95% CI = 0.92–5.31, lymph node involvement: adjusted HR = 5.94; 95% CI = 2.21–15.95) (Table 3).

**TABLE 2:** Univariate, age‐adjusted and multivariable‐adjusted Cox proportional hazards models for distant relapse in ER‐ breast cancers (*n* = 67).

Variable	Univariate	Age‐adjusted	Multivariable‐adjusted
	HR (95% CI)	HR (95% CI)	HR (95% CI)
Age	1.0 (0.97–1.03)	–	0.99 (0.95–1.02)
MCP‐1	2.00 (0.97–4.13)	2.00 (0.97–4.12)	1.66 (0.77–3.61)
Grade	0.57 (0.17–1.88)	0.57 (0.17–1.88)	0.56 (0.14–2.20)
Chemotherapy	1.00 (0.49–2.03)	0.96 (0.44–2.11)	0.77 (0.32–1.85)
Lymph node involvement	**4.92 (2.12–11.44)**	**5.06 (2.17–11.81)**	**4.91 (2.09–11.56)**

*Note*: Significant variables are depicted in bold (*p* < 0.05).

Abbreviations: CI, confidence interval; HR, hazard ratio; MCP‐1, monocyte chemoattractant protein‐1.

**TABLE 3:** Univariate, age‐adjusted and multivariable‐adjusted Cox proportional hazards models for breast cancer‐related death with distant relapse in ER‐ breast cancers (*n* = 67).

Variable	Univariate	Age‐adjusted	Multivariable‐adjusted
	HR (95% CI)	HR (95% CI)	HR (95% CI)
Age	0.98 (0.94–1.01)	–	0.97 (0.94–1.01)
MCP‐1	**2.84 (1.27–6.37)**	**2.96 (1.32–6.65)**	2.21 (0.92–5.31)
Grade	0.39 (0.09–1.64)	0.40 (0.10–1.71)	0.45 (0.09–2.33)
Chemotherapy	1.33 (0.62–2.86)	1.03 (0.43–2.43)	0.89 (0.34–2.33)
Lymph node involvement	**6.38 (2.41–16.88)**	**6.71 (2.53–17.81)**	**5.94 (2.21–15.95)**

*Note*: Significant variables are depicted in bold (*p* < 0.05).

Abbreviations: CI, confidence interval; HR, hazard ratio; MCP‐1, monocyte chemoattractant protein‐1.

### 3.5 Correlation of MCP‐1 expression in primary tumour with breast cancer stage

**FIGURE 4:**
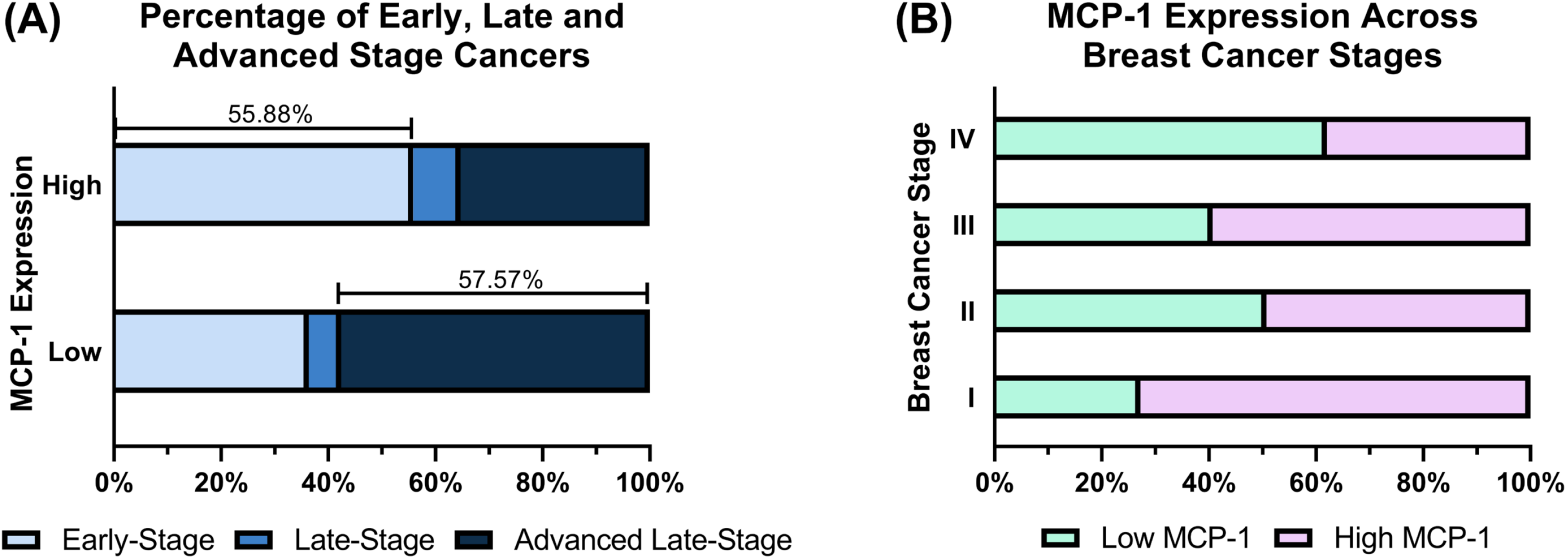
(A) Percentage of low MCP‐1 expression and high MCP‐1 expression groups that were early‐stage (Stage I and II), late‐stage (Stage III) and advanced late‐stage breast cancers (Stage IV). (B) Percentage of low MCP‐1 expression and high MCP‐1 expression cancers in Stage I, Stage II, Stage III and Stage IV cancers. These visual representations were adapted from Hofstee et al.^30^ monocyte chemoattractant protein‐1 (MCP‐1).

Subsequent to the association of low MCP‐1 expression with decreased survival, we wanted to explore the correlation of MCP‐1 with breast cancer stage, given breast cancer staging is a better indicator of cancer severity. High MCP‐1 expression in the primary tumour was correlated with Stage I breast cancers (*p* < 0.05; Table 4). Figure 4A shows that high MCP‐1 expressing tumours were predominantly early‐stage (Stage I and II; 55.88%) cancers, whereas low MCP‐1 expressing tumours were advanced late‐stage cancers (Stage IV; 57.57%). Figure 4B shows the variation in MCP‐1 expression across the different breast cancer stages. Stage I cancers predominantly expressed MCP‐1 highly, whereas Stage IV cancers predominantly expressed MCP‐1 lowly.

**TABLE 4:** Chi‐squared analysis of MCP‐1 expression and staging of ER‐ breast cancers to identify any potential association.

Breast cancer stage		Low (*n* = 33)		High (*n* = 34)		*χ* ^2^	*p*‐value
		Yes	No	Yes	No		
Stage I	Count (*n*)	4	29	11	23	3.945	**0.0470**
	% within group	12.12	87.88	32.35	67.65		
	% of total	5.97	43.28	16.42	34.33		
Stage II	Count (*n*)	8	25	8	26	0.005	NS
	% within group	24.24	75.76	23.53	76.47		
	% of total	11.94	37.31	11.94	38.81		
Stage III	Count (*n*)	2	31	3	31	0.4303	NS
	% within group	6.06	93.94	8.82	91.18		
	% of total	2.99	46.27	4.48	46.27		
Stage IV	Count (*n*)	19	14	12	22	1.829	NS
	% within group	57.58	42.42	35.29	64.71		
	% of total	28.36	20.90	17.91	32.84		

*Note*: Significant variables are depicted in bold (*p* < 0.05).

Abbreviations: MCP‐1, monocyte chemoattractant protein‐1; NS, not significant.

## 4 DISCUSSION

The use of MCP‐1 as a diagnostic marker for breast cancer metastasis has been seldom explored. Furthermore, the role of MCP‐1 in breast cancer and its use as a predictive tool remains conflicting. Importantly, the present study has shown that MCP‐1 expression in primary ER− breast tumours is varied across the four breast cancer stages in an Australian cohort of women.

Kaplan–Meier models identified ER− breast cancers as a predictor of distant relapse (Figure 2A) and breast cancer‐related death with distant relapse (Figure 2B). Given ER− breast cancers are generally more aggressive cancers, this association is not remarkable. When we looked at the ER− group further, low MCP‐1 expression in the primary tumour was associated with an increased risk of breast cancer‐related death with distant relapse. Currently, the literature is in overwhelming support of MCP‐1 as a pro‐metastatic chemokine–high levels of MCP‐1 have been associated with breast cancer stroma development,^11^, ^12^, ^13^, ^14^ angiogenesis,^15^, ^16^ progression,^8^, ^26^, ^27^ metastasis^17^, ^18^, ^19^ and poor prognosis–and so the association of low MCP‐1 expression with poor prognosis in this study is a surprising finding.

The usefulness of MCP‐1 as a prognostic marker in breast cancer has remained conflicted. Elevated levels of MCP‐1 in serum have been associated with advanced tumour stage and lymph node involvement,^20^ and also with favourable prognostic variables.^21^ Recently, Heiskala et al. suggested that a high number of MCP‐1‐positive primary breast tumour cells coupled with a high number of CD14‐positive TAMs was predictive of early relapse. Initial Kaplan–Meier analysis in the current study suggested that high MCP‐1 was favourable, implying that MCP‐1 is anti‐progression and anti‐metastatic, and would substantiate the findings of Dehqanzada that high MCP‐1 expression is associated with a positive prognostic outlook. It is important to consider here the method employed to assess MCP‐1 expression and the potential for discrepancies when interpreting data of this kind in the context of the available literature. Most studies investigating MCP‐1 and breast cancer either assess its expression using IHC or assess its levels in serum. This study assessed MCP‐1 expression by IHC, Dehqanzada assessed levels of MCP‐1 in serum. Future studies would benefit from considering both IHC expression and serum analysis to confirm whether associations of MCP‐1 expression and levels remain consistent across the investigatory methods. Nonetheless, the implication of MCP‐1 as an anti‐metastatic chemokine–as suggested by initial Kaplan–Meier analysis–would be inconsistent with the extensive literature associating MCP‐1 with disease progression. Instead, we hypothesised that MCP‐1 expression may drop in late and advanced late‐stage triple negative breast cancers that had already gained metastatic potential and, therefore, sought to reconsider our findings in the context of breast cancer stages.

Breast cancer stages consider tumour size, number of lymph nodes that are positive with cancer and incidence of metastasis or distant relapse together, and this can give a more robust indication of breast cancer aggression. Subsequent exploratory analysis indicated that high MCP‐1 expression was significantly correlated with Stage I ER− breast cancers (Table 4). In low MCP‐1 expressing ER− breast cancers, 57.57% were advanced late‐stage. Conversely, 55.88% of high MCP‐1 expressing primary ER− breast cancers were early‐stage (Figure 4A). If we consider this finding in the context of the literature supporting MCP‐1 as a pro‐progression, pro‐metastatic chemokine, our data paints a more detailed picture of MCP‐1 expression in the primary tumour across its progression. Patients with Stage I ER− breast cancers predominantly had high MCP‐1 expression (Figure 4B). If MCP‐1 is pro‐progression and pro‐metastatic, this finding supports this sentiment; the tumour is developing in its early stages and MCP‐1 expression is increased to optimise its progression. In this study, the cohort with the greatest risk of death with distant relapse was comprised of patients with predominantly Stage III and Stage IV ER− breast cancer (Figure 4A); however, what is particularly interesting, is the association of these patients with low MCP‐1 expression in the primary tumour.

In contrast to Stage I, patients with Stage IV ER− breast cancers predominantly showed low expression of MCP‐1 (Figure 4B). By definition, a Stage IV breast cancer must have evidence of distant relapse in another organ. This then, in turn, suggests that MCP‐1 expression may decrease in the primary tumour as it becomes less dominant, and the cancer becomes more prominent in other organs. A study on patients with salivary gland tumours highlighted a similar phenomenon, whereby MCP‐1 expression was lower in patients with advanced stage disease, citing that this low expression likely confirmed inadequate recruitment of the mononuclear inflammatory cells necessary to mount an effective anti‐tumour cytotoxic response.^28^ It is important to acknowledge here that the sample size of this study is relatively small, and these findings need to be corroborated in a larger study; future studies may consider in‐silico analysis of existing, publicly available cancer datasets to substantiate their findings. Additionally, examining MCP‐1 expression in circulating breast tumour cells as well as at the metastatic site may be useful in elucidating MCP‐1's role in breast cancer progression. Furthermore, tracking MCP‐1 expression in breast cancer patients as their disease progresses might also provide important insights into MCP‐1 s role in metastasis. Nonetheless, if these findings are corroborated, it will be of particular importance to elucidate whether this switch from high to low expression of MCP‐1 in the primary tumour is a by‐product of the tumour having metastasised, or whether the switch is mechanistic and may drive, in part, the metastasis of the tumour. If it is the latter, then anti‐MCP‐1 therapies—such as the use of propagermanium^29^—delivered to patients with ER− breast cancers may be harmful and afford their cancer metastatic potential.

These data highlight an inherent need for studies of this nature to consider their findings in the context of breast cancer stages. Most studies will assess tumour size, grade, lymph node involvement and metastasis as separate variables. In the clinical setting, we consider these variables together to give a more descriptive status of the cancer. It is important that studies assessing potential prognostic markers also consider how these variables may interact as one. Cox proportional hazards models (Tables 2 and 3) highlighted that MCP‐1 expression was likely confounded by lymph node involvement. If we then consider this in the context of breast cancer stages, this confounder is not surprising. Most ER− breast cancers with low expression were of late or advanced late‐stage (Stage III or Stage IV cancers). By definition, for a breast cancer to be considered Stage III, it must have substantial lymph node involvement and almost all cancers that had reached Stage IV had strong evidence of lymph node involvement. Again, it appears that the most telling variable in this study is the one that considers all factors related to the primary tumour as one: breast cancer stage; and, as such, highlights the importance of considering breast cancer stages when interpreting data of this kind. Future studies should seek to further characterise MCP‐1 expression in Stage I, II, III and IV breast cancers across multiple subtypes and elucidate the difference in potential of early‐stage tumours with low and high expression of MCP‐1.

## 5 CONCLUSIONS

This study has highlighted the innate complexity of MCP‐1's role in breast cancer progression and metastasis. Low MCP‐1 expression in the primary tumour was associated with breast cancer‐related death with distant relapse in ER− breast cancers; however, this was likely a result of those cancers being Stage III or Stage IV. As such, we highlight the importance of studies considering their results in the context of breast cancer stages. Importantly, our data shows a variation in expression of MCP‐1 in primary ER− tumours across the stages of breast cancer, with the highest expression in Stage I tumours and the lowest expression in Stage IV tumours. MCP‐1 may still have value as a prognostic variable and is still a valid therapeutic target, particularly for adjuvant chemokine therapy, but what has been made acutely evident from the present study, is that progression in this field needs to be navigated with extreme caution.

## AUTHOR CONTRIBUTIONS

**Bridie S. Mulholland:** Conceptualization (lead); formal analysis (lead); funding acquisition (lead); investigation (lead); methodology (equal); project administration (lead); writing – original draft (lead); writing – review and editing (equal). **Pierre Hofstee:** Conceptualization (supporting); formal analysis (supporting); investigation (supporting); methodology (supporting); project administration (supporting); writing – original draft (supporting); writing – review and editing (equal). **Ewan K.A Millar:** Data curation (equal); formal analysis (supporting); investigation (supporting); methodology (supporting); writing – review and editing (equal). **Dana Bliuc:** Formal analysis (supporting); investigation (supporting); methodology (supporting); writing – review and editing (equal). **Sandra O'Toole:** Data curation (equal); resources (lead); writing – review and editing (equal). **Mark R Forwood:** Conceptualization (supporting); funding acquisition (supporting); investigation (supporting); project administration (supporting); resources (supporting); writing – review and editing (equal). **Michelle M McDonald:** Conceptualization (supporting); investigation (supporting); methodology (supporting); project administration (supporting); resources (supporting); writing – review and editing (equal).

## FUNDING INFORMATION

B.S.M was provided funding for this research from the School of Pharmacy and Medical Sciences at Griffith University. The funding source did not have any input into the design, conduct, analysis or interpretation of the study, or the production of this manuscript. EKAM is supported by a Researcher Exchange and Development in Industry (REDI) Fellowship from MTPConnect/MRFF Australia.

## CONFLICT OF INTEREST STATEMENT

The authors declare that they have no competing interests.

## ETHICS STATEMENT

Ethics for this study was obtained from the St Vincent's Hospital Human Research Ethics Committee (HREC SVH H94/080; HREC 06336 SVH H00 036). All breast tumour tissue samples were obtained between February 1992 and August 2002.

## Data Availability

The data that support the findings of this study are available from the Garvan Institute of Medical Research, but restrictions apply to the availability of these data, which were used under license for the current study, and so are not publicly available. Data are however available from the authors upon reasonable request and with permission of the Translational Breast Cancer Research Group at the Garvan Institute of Medical Research.
